# Supplementary material for: Elimination of Airborne Microorganisms Using Compressive Heating Air Sterilization Technology (CHAST): Laboratory and Nursing Home Setting
Source: Microorganisms. 2025 Oct 3;13(10):2299. doi: 10.3390/microorganisms13102299 (PMC12566561; doi:10.3390/microorganisms13102299)
Supplement: Supplementary file 1 [file microorganisms-13-02299-s001.zip › microorganisms-3814792-supplementary.pdf]

Supplementary Materials for Elimination of Airborne Microorganisms Using Compressive Heating Air Sterilization Technology (CHAST): Laboratory and Nursing Home Setting.

Pritha Sharma, Supriya Mahajan, Gene D. Morse, Rolanda L. Ward, Satish Sharma, Stanley A. Schwartz and Ravikumar Aalinkeel

Energy and Cost Balance Evaluation:

This supplementary section provides a detailed analysis of the energy and cost balance of the Compressive Heating Air Sterilization Technology (CHAST). The analysis is performed using first-order thermodynamic calculations and compared side-by-side with common market alternatives such as portable HEPA filters and ultraviolet germicidal irradiation (UVGI) systems.

Methodology

The energy intensity of CHAST is determined by using a first-order model of thermal load per cubic meter of processed air. The specific heat capacity of air (cp), air density (ρ), and temperature rise (ΔT) from 20 °C to 240 °C are considered. With a counterflow heat exchanger of 85–90% effectiveness, the net thermal energy requirement per unit volume is significantly reduced. Fan power requirements are also considered but remain small compared to the heating load.

First-order Model Equations

Thermal load per m^3 of air (no heat recovery):

$E = \rho \times cp \times \Delta T$

$= 1.2\ kg/m^3 \times 1.005\ kJ/kg{\cdot}K \times (240 – 20)\ K$

$= 265\ kJ/m^3 \approx 0.0737\ kWh/m^3$.

With a counterflow heat exchanger of effectiveness (ηHX = 0.85–0.90):

Net E = 0.0111–0.00737 kWh/m^3.

Fan power (typical):

$P \approx \Delta P \times Q / \eta f$

For ~1 in w.g. (≈250 Pa) and ηf ≈ 0.6, fan energy is small compared with heating requirements.

Side-by-side Comparison with Alternatives

Technology	Energy Intensity (kWh/m^3)	Notes
**CHAST (with heat recovery)**	0.007–0.011	High-efficiency sterilization, >6-log reduction
**HEPA Filtration**	0.003–0.005	Particle filtration only, no sterilization
**UVGI**	0.004–0.006	Dependent on lamp life and maintenance

Cost Analysis

Using the U.S. commercial electricity price of approximately $0.13/kWh (EIA Table 5.3, June 2025) [1–3], the effective cost per cubic meter of sterilized air with CHAST ranges between $0.00096 and $0.0014. This compares favorably with UVGI and HEPA filtration when normalized for sterilization efficiency, as CHAST uniquely achieves complete microbial inactivation rather than simple particle removal or partial reduction.

When CHAST Can Be Worth the Higher Energy

- Risk profile:

Laboratory and scaled tests show per-pass ≥6-log reduction (and >7-log at 5000 CFM) across spores, vegetative bacteria, and MS2 virus, demonstrating sterilization of the airstream before discharge. This capability is extremely important in settings where capture/inactivation uncertainty is unacceptable, such as isolation rooms, procedure rooms, or during outbreaks.

- Byproducts/consumables:

No filters or UV lamps require replacement, minimizing waste streams. However, electricity remains the dominant operating expense.

- Scalability:

Efficacy has been demonstrated at flow rates from 300–5000 CFM with maintained log-reductions. Energy scales linearly with flow and inversely with heat-recovery effectiveness.

Bottom Line (Balanced View)

- Energy/cost:

CHAST exhibits higher energy intensity than HEPA and typically somewhat higher than well-designed UVGI on a per-unit air handled basis.

- Performance:

In return, CHAST provides per-pass sterilization with consistently high log-reductions across a range of organisms and flow scales. This performance can justify the operational expense in high-risk, mission-critical zones. In other contexts, HEPA and/or UVGI offer far lower energy costs and are appropriate as primary layers within a hybrid strategy.

References

1. U.S. Energy Information Administration (EIA). Electric Power Monthly, Table 5.3, June 2025.
2. Kowalski W. Ultraviolet Germicidal Irradiation Handbook. Springer; 2009.
3. ASHRAE Standard 52.2-2017: Method of Testing General Ventilation Air-Cleaning Devices.

Detailed results for Evaluating the Efficacy of CHAST Under Controlled Laboratory Conditions

(i) Early Results and Proof-of-Concept Experiments: Initial proof-of-concept testing was conducted at the University at Buffalo using an early prototype of the CHAST system. In this study, air containing *Bacillus globigii* (Bg), a non-pathogenic surrogate for *Bacillus anthracis*, was passed through the system at 247°C. A >3-log (99.9%) reduction in viable spores was observed, confirming initial sterilization efficacy (Supplemental Table 1, Row 1). Subsequent studies were done at the Department of Defense (DoD) and tested the same prototype against Bst, a highly heat-resistant organism, under similar conditions but at 240°C. These tests achieved >6-log (99.9999%) reduction in viable bacteria (Supplemental Table 1, Row 2). Further, an improved CHAST system (300 CFM GD 450 unit) was assessed against Bg, Bt, and the MS2 bacteriophage, and the results showed consistent >6-log (99.9999%) kill rates across all tested organisms (Supplemental Table 1, Row 3). Next, a modified 300 CFM GD 540 CHAST system was tested at 240°C under varied flow conditions, and the results again demonstrated >6-log reductions against Bg (Supplemental Table 1, Row 4). In another set of tests, when a 300 CFM GD (HF-408) unit with a tri-lobe PD blower was used, the results again showed >6-log reductions against Bg, *E. coli*, and MS2 bacteriophage (Supplemental Table 1, Row 5). In the next set of experiments, with an enhanced version of the same unit incorporating a high effectiveness counterflow heat exchanger was evaluated. The results showed identical results against Bg as the improved CHAST system (Supplemental Table 1, Row 6). We later evaluated the Alpha prototype (300 CFM GD HF-408) for its efficacy in pathogen removal, and the results demonstrated >6-log reduction against Bg at 240°C under varying flow and temperature conditions (Supplemental Table 1, Row 7). An optimized version of the same system also confirmed this performance (Supplemental Table 1, Row 8). Furthermore, a high-capacity 1000 CFM centrifugal counterflow unit tested at 220°C maintained the same >6-log kill efficacy (Supplemental Table 1, Row 9). To support large-scale deployment, the system was scaled up into a 5000 CFM mobile prototype, and tests using this model also confirmed sustained efficacy against Bg spores at 240°C, with >7-log (99.99999%) inactivation and no detectable viable spores downstream (Supplemental Table 1, Row 10).

Legend to Supplemental Table S1: This table summarizes laboratory testing results evaluating the microbial inactivation efficacy of various CHAST prototypes operating under controlled thermal conditions. Aerosolized biological agents, including *Bacillus globigii (Bg), Bacillus stearothermophilus* (Bst), *Bacillus thuringiensis* (Bt), *Escherichia coli*, and MS2 bacteriophage, were introduced at known concentrations upstream of the CHAST unit inlet. Treated and untreated air samples were collected via impingers and cultured under organism-specific conditions to determine viable counts. Kill efficacy was determined by comparing colony-forming units (CFU) or plaque-forming units (PFU) recovered from inlet (baseline) versus outlet (treated) samples. Log reduction values ($\log_{10}$) were calculated using the formula: Percent Reduction = [(Mean Baseline CFU − Mean Treated CFU) / Mean Baseline CFU] × 100. All prototypes achieved ≥99.9% kill under high-temperature operation (≥220°C), with most systems demonstrating >6-log (99.9999%) reduction, confirming CHAST's robust thermal sterilization capability across multiple flow configurations.

Supplemental Table S1. CHAST Kill Efficacy Under Varying Conditions Against Airborne Microorganisms

Test Objective(s)	Temperature (°C)	Challenge Organism(s)	Log or % Kill Rate	Unit / Prototype Description
**Kill efficacy against bacteria**	247	*Bacillus globigii (Bg)*	>3-log (99.9%)	Initial prototype, University at Buffalo
**Kill efficacy against bacteria**	240	*Bacillus stearothermophilus (Bst)*	>6-log (99.9999%)	Initial prototype, DoD
**Kill efficacy against bacteria and virus**	240	*Bacillus globigii (Bg), Bacillus thuringiensis (Bt), MS2 bacteriophage*	>6-log (99.9999%)	300 CFM (GD 450), Research Triangle Institute
**Kill efficacy against bacteria**	240	*Bacillus globigii (Bg)*	>6-log (99.9999%)	300 CFM (GD 540), varied flow rates and temperature, DoD 2006 Program
**Kill efficacy against bacteria, E. coli, and virus**	240	*Bacillus globigii (Bg), E. coli, MS2 bacteriophage*	>6-log (99.9999%)	300 CFM (HF-408), tri-lobe PD blower, DoD 2006 Program
**Kill efficacy against bacteria**	240	*Bacillus globigii (Bg)*	>6-log (99.9999%)	300 CFM (HF-408), tri-lobe PD blower + counterflow heat exchanger, DoD 2006 Program
**Kill efficacy against bacteria**	240	*Bacillus globigii (Bg)*	>6-log (99.9999%)	300 CFM (HF-408), Alpha prototype, varied flow and temperature, DoD 2009 Program
**Kill efficacy against bacteria**	240	*Bacillus globigii (Bg)*	>6-log (99.9999%)	300 CFM (HF-408), optimized prototype, DoD 2009 Program
**Kill efficacy against bacteria**	220	*Bacillus globigii (Bg)*	>6-log (99.9999%)	1000 CFM, centrifugal counterflow, DoD 2009 Program
**Maintenance of kill efficacy (scaled system)**	240	*Bacillus globigii (Bg)*	>7-log (99.99999%)	5000 CFM, centrifugal counterflow, DoD SBIR Program
